# The associations between workplace bullying, salivary cortisol, and long-term sickness absence: a longitudinal study

**DOI:** 10.1186/s12889-017-4716-7

**Published:** 2017-09-16

**Authors:** Matias Brødsgaard Grynderup, Kirsten Nabe-Nielsen, Theis Lange, Paul Maurice Conway, Jens Peter Bonde, Anne Helene Garde, Maria Gullander, Linda Kaerlev, Roger Persson, Reiner Rugulies, Marianne Agergaard Vammen, Annie Høgh, Åse Marie Hansen

**Affiliations:** 1 Department of Public Health, Centre for Health and Society, University of Copenhagen, Øster Farimagsgade 5, 1014 Copenhagen K, Denmark; 2 Center for Statistical Science, Peking University, No.5 Yiheyuan Road Haidian District, 100871 Beijing, People’s Republic of China; 3 Department of Psychology, University of Copenhagen, Øster Farimagsgade 2A, 1353 Copenhagen K, Denmark; 4 Department of Occupational and Environmental Medicine, Frederiksberg and Bispebjerg Hospital, Bispebjerg Bakke 23, 2400 Copenhagen, NV Denmark; 5 The National Research Centre for the Working Environment, Lersø Parkalle 105, 2100 Copenhagen Ø, Denmark; 6 Research Unit of Clinical Epidemiology, Institute of Clinical Research, University of Southern Denmark, Campusvej 55, 5230 Odense M, Denmark; 7 Center for Clinical Epidemiology, Odense University Hospital, Sdr. Boulevard 29, 5000 Odense C, Denmark; 8 Department of Psychology, Lund University, Box 213, 221 00 Lund, Sweden; 9 Division of Occupational and Environmental Medicine, Lund University, SUS, 221 85 Lund, Sweden

**Keywords:** Cortisol, Mediation, Saliva, Sickness absence, Workplace bullying

## Abstract

**Background:**

Workplace stressors, such as bullying, are strongly related to subsequent long-term sickness absence, but little is known of the possible physiological mechanisms linking workplace stressors and sickness absence. The primary aim of this study was to investigate to what extent cortisol levels were associated with subsequent sickness absence and if cortisol mediated the association between workplace bullying and sickness absence. We additionally investigated possible bidirectional associations between bullying, cortisol, and long-term sickness absence.

**Methods:**

Participants came from two Danish cohort studies, the “Psychosocial RIsk factors for Stress and MEntal disease” (PRISME) cohort and the “Workplace Bullying and Harassment” (WBH) cohort (*n* = 5418). Information about exposure to workplace bullying and morning and evening salivary cortisol was collected at three time points with approximately two years in between. After each data collection, all participants were followed for two years in registers, and cases with long-term sickness absence lasting 30 or more consecutive days were identified. The association between cortisol levels and subsequent sickness absence was assessed by logistic regression, while the extent to which the association between bullying and sickness absence was mediated by cortisol was quantified through natural direct and indirect effects.

**Results:**

High evening cortisol was associated with a decreased risk of sickness absence (OR = 0.82, 95% CI = 0.68–0.99), but we did not find that high morning cortisol levels (OR = 0.98, 95% CI = 0.81–1.18) or high morning-to-evening slope (OR = 0.99, 95% CI = 0.82–1.18) were associated with subsequent sickness absence. We also tested for reverse causation and found that long-term sickness absence, but not salivary cortisol, was a strong risk factor for subsequent workplace bullying. There was no indication that cortisol mediated the association between workplace bullying and sickness absence.

**Conclusion:**

We found no straightforward and simple association between cortisol and long-term sickness absence. Furthermore, the association between workplace bullying and long-term sickness absence was not mediated by cortisol.

## Background

Sickness absence can have large personal costs and negative effects for the absent, but also have extensive costs to the society [1]. The causes of sickness absence are multifactorial and while prolonged periods of sickness absence are highly reflective of poor health, other factors, such as behavior, may also play an important role [2]. Several studies have shown that psychosocial work factors are related to sickness absence, and bullying is one of the factors that are most strongly related to sickness absence [3–7]. An important question is through which mechanisms workplace bullying causes sickness absence. The association between workplace bullying and sickness absence is partially mediated by sleep problems [8] and perceived stress [9] and other factors, such as coping strategies, behavior, and physiology may also be important.

Increased activation of the HPA-axis and subsequent changes in cortisol secretion has been suggested as a biological pathway linking psychological stressors to somatic diseases [10]. The association between cortisol and specific health outcomes, such as cardiovascular disease, cancer, pain, anxiety, and depression, have been examined in several studies [11, 12]. While the results of previous research are not entirely consistent, the majority of studies indicate that low levels of cortisol in the morning and high levels in the evening are related to poor somatic health, but not to mental health [11, 12].

Several cross-sectional studies have shown that bullied persons tend to have lower diurnal circulating cortisol concentrations as assessed with saliva sampling [13–16]. Whether this reflects adrenal insufficiency and a reduced capacity to produce cortisol is not known. However, since acute stress is believed to increase HPA-axis activity and that chronic stress in conditions such as the chronic fatigue syndrome is associated with lower HPA-axis activity [17], it seems plausible to assume that: 1) early stages of workplace bullying are associated with higher cortisol levels and 2) a shift towards lower cortisol levels occurs when the bullying experience progresses to be perceived as very severe by the target. However, the empirical evidence supporting these assumptions is scarce and no study seems to have investigated whether cortisol levels are indicative of future periods of long-term sickness absence. Likewise, no studies have yet examined if the association between bullying and sickness absence is mediated by cortisol levels.

The primary aim of this follow-up study was therefore to investigate, in a large cohort of Danish employees, to what extent morning or evening cortisol levels were associated with long-term sickness absence and if cortisol mediated the association between workplace bullying and long-term sickness absence. We hypothesized that participants with lower morning and higher evening cortisol concentrations would exhibit a higher occurrence of long-term sickness absence, and that the association between workplace bullying and subsequent long-term sickness absence would be partially mediated by decreased cortisol concentrations. We additionally investigated possible bidirectional associations between bullying, cortisol, and long-term sickness absence.

## Methods

### Study population

**Fig. 1:**
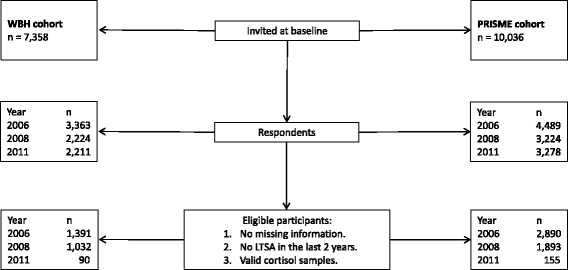
Outline of study population. Individuals invited at baseline, respondents, and eligible participants by cohort and rounds

This follow-up study is based on two Danish cohorts, the “Psychosocial RIsk factors for Stress and MEntal disease” (PRISME) and the “Workplace Bullying and Harassment” (WBH) cohorts. The PRISME cohort was established in 2007 and reexamined in 2009 [18]. A total of 10,036 civil servants and hospital employees from the municipality of Aarhus and the Central Denmark Region were invited for the first round of the study, and 4489 (45%) participated by collecting saliva samples and questionnaire information. All respondents from the first round were re-invited and 3224 (72%) participated in the second round. The WBH cohort was established in 2006 and reexamined in 2008 [19]. A total of 7358 employees from public and private workplaces across Denmark were invited for the first round, and 3363 (46%) participated by collecting saliva samples and questionnaire information. All respondents from the first round were invited again in addition to a small group of newly invited (*n* = 338), and 2224 (60%) participated in the second round. The two cohorts were merged and all participants who were invited in the second round of the PRISME or WBH study were re-invited a third time in 2011 [19]. A total of 3278 (73%) participated from the PRISME cohort and 2211 (60%) from the WBH cohort. All those invited in the third round of the study received a questionnaire. Based on the questionnaire responses we invited all bullied participants (*n* = 428) and a random subsample of non-bullied participants (*n* = 364) to collect saliva samples. Two to six months after sending out the questionnaire we obtained saliva samples from 161 bullied (38%) and 148 non-bullied participants (41%). In total 8408 unique participants took part in one or more of the study rounds. We excluded all observations with missing information on one or more of the variables used in the analysis. To avoid reverse causation, we excluded all observations with long-term sickness absence (LTSA) within the last two years from each round of the study. Participants that did not collect their cortisol samples within a specific time-window, as described later, were also excluded. A total of 5418 eligible participants contributed with 7451 observations across the three rounds of the study (Fig. 1).

### Sickness absence

Information on sickness absence was obtained by linkage to the Danish register of sickness absence compensation benefits and social transfer payments (RSS) [20]. Currently, all Danish employers are entitled to sickness absence compensation for employees that are absent for 30 or more continuous days and such cases of sickness absence are registered in the RSS. We followed all participants in the RSS and recorded periods of sickness absence of 30 days or more up to 2 years after they filled in the questionnaire. Participants who took part in more than one round of the study were followed for a two-year period after every round in which they participated. Of the 7451 two-year periods followed up in the RSS, a total of 842 included one or more spells of sickness absence with a duration of 30 or more days. We also obtained information about long-term sickness absence for non-participants in the study to examine if the risk of long-term sickness absence was different between participants and non-participants.

### Measures of workplace bullying

Bullying was assessed with one question in all three rounds of the study: “Have you been subjected to bullying at work within the past 6 months?” with the following response categories: never, now and then, monthly, weekly, and daily. In the third round of the study bullying was instead assessed within the past 12 months. The question was preceded by the following definition: “Bullying occurs when one or more persons repeatedly over a longer period, are exposed to unpleasant or negative behavior at work that it is difficult to defend one-self against.” Participants who answered never were defined as non-bullied and participants who answered ‘now and then’, ‘monthly’, ‘weekly’, or ‘daily’ were defined as bullied, while only participants reporting bullying ‘weekly’ or ‘daily’ were defined as frequently bullied. The primary analyses relied on all bullied participants, while the frequently bullied were only examined in sensitivity analyses.

### Measures of cortisol

In the first two rounds of the study all participants were instructed to provide saliva samples in Salivette® tubes containing cotton swaps that were delivered along with the questionnaire. The participants were instructed to keep the swap in their mouth until saturated and to return the tubes by mail after sampling. In the third round the participants collected saliva samples by spitting directly into Salivette® without swabs. Cortisol exhibits a distinct diurnal variation. This pattern offers several challenges when selecting a sampling strategy. Each participant was asked to provide two samples, the first in the morning 30 min after awakening, and the second in the evening at 20:00 h. For the morning sample, the aim was to detect the morning cortisol peak that is expected to occur about 30–45 min after awakening [21, 22]. Because the cortisol concentration declines slowly during the day and is stable during the evening [23, 24] sampling time is less important in the evening and we decided on a fixed time. We also calculated the difference between the morning and evening cortisol concentrations, called the slope or diurnal variation, which indicates the daily capacity for recovery [11]. The participants were instructed to keep the samples stored in a refrigerator until they were returned by mail to the research institution. The samples were then stored at −20 °C and analyzed within a year.

In the first two rounds the measurement of cortisol concentration in the saliva samples was carried out with the Spectria Cortisol Coated Tube radioimmunoassay (Orion Diagnostica, Espoo, Finland). In the third round the measurements were carried out with a liquid chromatography tandem mass spectrometry method (LC-MSMS) [25]. Concentrations below the limit of detection were assigned a random value between 0 and the limit of detection using a uniform distribution. Concentrations from the third round were adjusted to account for differences in sampling and measurement methods [26, 27] compared to the first two rounds. We performed a method comparison by analyzing 100 samples with both the immunoassay and LC-MSMS methods. The LC-MSMS results were comparable to the immunoassay by adding 1.19 nmol/l to the LC-MSMS concentration and then multiplying it by 1.76 (unpublished data). Morning and evening cortisol samples were considered valid only if they were collected within the first two hours after awakening and between 16:00 and 04:00 h, respectively. Concentrations above 100 nmol/l were likely to occur due to measurement error, and were therefore excluded. Morning-to-evening slope were calculated by subtracting the evening cortisol concentration from the morning cortisol concentration.

### Covariates

The following covariates were selected due to being risk factors of sickness absence [28–31] or predictors of cortisol levels [32–34] and were included in all analyses: gender, age (continuous), smoking (current, former, never), physical activity (<2, ≥2 h per week), alcohol consumption (≤14, >14 g per week), body mass index (<18.5, 18.5–25, >25 kg/m2), education (<3, 3–4, >4 years beyond primary or high school), shift-work (day, evening, night, rotation). Furthermore, we adjusted for round (1st round, 2nd round, 3rd round), and cohort (PRISME, WBH) in order to take differences between rounds and cohort into account. Analyses of cortisol was further adjusted for time of awakening (continuous) and sampling time (continuous and squared).

### Data for the reverse causation analyses

To examine any potential reverse causation between workplace bullying, sickness absence and salivary cortisol we needed to restructure the dataset. Since we only had registry information about sickness absence, and not workplace bullying or salivary cortisol levels, we needed a distinct follow-up examination to measure these factors. In our three-round study, we were able to construct two separate courses, one course with baseline information from round 1 and follow-up information from round 2, and one course with baseline information from round 2 and follow-up information from round 3. We pooled information from the two courses, and used this dataset in all reverse causation analyses. In the analyses of cortisol as a predictor of workplace bullying and sickness absence as a predictor of workplace bullying we excluded all participants reporting workplace bullying at baseline. In the analysis of the influence of sickness absence on cortisol concentrations, we excluded all participants with a history of sickness absence in the last two years before baseline.

### Statistical analyses

#### Bullying and long-term sickness absence

The association between bullying and sickness absence was assessed by logistic regression. As each individual contributed data up to three times all analyses were performed with robust standard errors based on clusters formed by the individual participants. Bullying and long-term sickness absence were included as dichotomous variables (yes, no) in all analyses.

#### Bullying and cortisol

The association between bullying and cortisol concentration was assessed by linear regression. We included morning and evening cortisol as logarithmically transformed continuous variables to normalize cortisol distribution, while morning-to-evening slope was not transformed.

#### Cortisol and long-term sickness absence

The association between cortisol concentration and sickness absence was assessed by logistic regression. Morning cortisol, evening cortisol, and morning-to-evening slope were included as categorical variables based on tertiles (low, medium, high) to account for a possible non-linear association between cortisol and bullying or long-term sickness absence.

#### Mediation

The extent to which the association between bullying and sickness absence was mediated by cortisol was quantified through natural direct and indirect effects [35, 36], which measures how large an effect we observe if bullying had no effect on cortisol (the natural direct effect) and if bullying only had effect on sickness absence through its effect on cortisol (the natural indirect effect). Estimation was done using the medflex package for R [37] with the imputation option for the estimation and bootstrap for standard errors. Bullying and sickness absence were included as dichotomous variables in the mediation analysis, while morning cortisol, evening cortisol, and morning-to-evening slope were included as 3-level categorical variables based on tertiles.

#### Reverse causation

To examine any potential reverse causation between workplace bullying, sickness absence and salivary cortisol we used the dataset created specifically for these analyses. We used logistic regression to examine if cortisol levels (categorized in tertiles) predicted subsequent workplace bullying in a population of participants, who did not report bullying at baseline. We used linear regression to examine whether changes in cortisol levels from baseline to follow-up were different among participants with and without episodes of long-term sickness absence during the follow-up period. Finally, we used logistic regression to examine if the two-year history of long-term sickness absence among participants not reporting bullying at baseline were a risk factor for workplace bullying at follow-up.

#### Sensitivity analyses

To examine the effects of more frequent bullying, we also performed a sensitivity analysis where we compared the participants that reported being bullied ‘daily’ or ‘weekly’ to the participants reporting never being bullied.

To examine if we needed to include interaction effects in the analyses, we used logistic regression to estimate the moderating effect of cortisol on the associations between workplace bullying and sickness absence by testing for multiplicative interaction.

To examine the effect of differential participation in our study, we compared baseline measures of workplace bullying and cortisol levels between participants and non-participants at follow-up. We also examined the association between study participation and long-term sickness absence at both baseline and follow-up.

The mediation analyses were performed in R studio version 3.2.0. All other analyses were conducted with the STATA 13 statistical software.

## Results

The characteristics of the study participants are described in Table 1 and are based on all 7451 observations included in the statistical analyses. We identified 430 participants who reported bullying in at least one round of the study. Since several participants reported bullying in more than one round, we identified 565 observations where bullying was reported across all three rounds of the study. The baseline characteristics of the study population are reported in Table 1.

**Table 1:** Characteristics of participants based on workplace bullying at start of follow-up and long-term sickness absence during the follow-up period based on 7451 observations from 5418 unique participants

	Long-term sickness absence			
	No long-term sickness absence during follow-up		Long-term sickness absence during follow-up	
	n	%	n	%
Gender				
Men	1729	26.2	144	17.1
Women	4880	73.8	698	82.9
Age				
< 35 years	1162	17.6	117	13.9
35–44 years	1763	26.7	202	24.0
45–54 years	2146	32.5	295	35.0
> 55 years	1538	23.3	228	27.1
Smoking				
Current	926	14.0	154	18.3
Former	2281	34.5	310	36.8
Never	3402	51.5	378	44.9
Alcohol consumption				
≤ 14 g per week	4872	73.7	654	77.7
> 14 g per week	1737	26.3	188	22.3
Physical activity				
< 2 h per week	718	10.9	114	13.5
≥ 2 h per week	5891	89.1	728	86.5
Body mass index				
< 18.5 kg/m^2^	111	1.7	14	1.7
18.5–25 kg/m^2^	4136	62.6	447	53.1
> 25 kg/m^2^	2362	35.7	381	45.3
Education				
< 3 years	1660	25.1	261	31.0
3–4 years	3899	59.0	513	60.9
> 4 years	1050	15.9	68	8.1
Bullying				
Non-bullied	6148	93.0	738	87.7
Bullied	461	7.0	104	12.4
Morning cortisol				
Low morning cortisol	2097	31.7	275	32.7
Moderate morning cortisol	2230	33.7	278	33.0
High morning cortisol	2282	34.5	289	34.3
Evening cortisol				
Low evening cortisol	2104	31.8	280	33.3
Moderate evening cortisol	2186	33.1	299	35.5
High evening cortisol	2319	35.1	263	31.2
Morning-to-evening slope				
Low slope	2155	32.6	281	33.4
Moderate slope	2227	33.7	277	32.9
High slope	2227	33.7	284	33.7

In the analysis of differential participation, we found that participants who were exposed to workplace bullying at baseline or had low baseline cortisol levels were less likely to participate in the follow-up rounds. Non-participants at baseline and follow-up also had an increased risk of sickness absence compared to the participants of the study (data not shown).

We found no indication that cortisol levels moderated the association between workplace bullying and subsequent sickness absence or the association between sickness absence and subsequent workplace bullying (*p* > 0.1 in all cases). Thus, we did not include any interaction effects in our analyses.

Table 2 shows the prospective associations of cortisol at baseline with long-term sickness absence during two years of follow-up. Neither morning cortisol nor the morning-to-evening slope was associated with risk of sickness absence. High evening cortisol was associated with a decreased risk of sickness absence (Odds ratio (OR) = 0.82, 95% confidence interval (CI) = 0.68–0.99).

**Table 2:** Prospective associations from a logistic regression analysis of cortisol at baseline with long-term sickness absence during two years of follow-up

	Sickness absence^a^	No sickness absence^b^	Crude OR	95% CI	Adjusted^c^ OR	95% CI
Morning cortisol						
Low (0–9.8 nmol/l)	275	2097	1	–	1	–
Moderate (9.9–15.8 nmol/l)	278	2230	0.95	0.80–1.14	0.97	0.80–1.16
High (15.9–100 nmol/l)	289	2282	0.97	0.81–1.15	0.98	0.81–1.18
Evening cortisol						
Low (0–1.0 nmol/l)	280	2104	1	–	1	–
Moderate (1.1–1.8 nmol/l)	299	2186	1.03	0.86–1.22	1.03	0.86–1.23
High (1.9–100 nmol/l)	263	2319	0.85	0.71–1.02	0.82	0.68–0.99
Morning-to-evening slope						
Low (−100–8.1 nmol/l)	281	2155	1	–	1	–
Moderate (8.2–14.2 nmol/l)	277	2227	0.95	0.80–1.14	0.97	0.81–1.16
High (14.3–100 nmol/l)	284	2227	0.98	0.82–1.17	0.98	0.82–1.18

^a^Number of participants with long-term sickness absence during follow-up distributed by cortisol levels

^b^Number of participants with no long-term sickness absence during follow-up distributed by cortisol levels

^c^Adjusted for gender, age, smoking, physical activity, alcohol consumption, body mass index, education, shift-work, awakening time, sampling time, round, and cohort. All measured at baseline

Table 3 shows the association of workplace bullying with cortisol concentrations. We found no association between workplace bullying and morning cortisol levels, evening cortisol levels, or morning-to-evening slope.

**Table 3:** Comparison of morning cortisol, evening cortisol, and morning-to-evening slope among bullied and non-bullied participants

	Cortisol concentrations^a^		Difference in cortisol concentration between bullied and non-bullied participants^b^			
	Bullied participants	Non-bullied participants	Crude β	95% CI	Adjusted^c^ β	95% CI
Morning cortisol^d^	2.51	2.48	0.03	−0.05-0.09	0.02	−0.03-0.08
Evening cortisol^d^	0.33	0.36	−0.03	−0.10-0.04	−0.06	−0.12 − 0.01
Morning-to-evening slope^e^	12.76	12.06	0.70	-0.01-1.40	0.53	−0.18-1.24

^a^Mean cortisol concentrations for bullied and non-bullied participants

^b^Results from a linear regression analysis

^c^Adjusted for gender, age, smoking, physical activity, alcohol consumption, body mass index, education, shift-work, awakening time, sampling time, round, and cohort. All measured at baseline

^d^Cortisol concentration (nmol/l) on the logarithmic scale

^e^Difference between morning and evening cortisol (nmol/l) of bullied participants compared to non-bullied participants

Table 4 shows that workplace bullying was significantly associated with subsequent sickness absence during a two-year follow-up period with an adjusted OR of sickness absence for bullied compared to non-bullied participants of 1.85 (1.47–2.33). The mediation analyses showed that the association between bullying and sickness absence was not mediated by any of the measures of cortisol. For all three cortisol measures, the proportion of the total association between bullying and sickness absence that was mediated by cortisol levels was less than 1% with very narrow confidence intervals indicating that any effect is not only insignificant, but null for all intents and purposes.

**Table 4:** Total, direct, and indirect effects of bullying on long-term sickness absence when including morning cortisol, evening cortisol, and morning-to-evening slope as potential mediators

	Mediator: Morning cortisol		Mediator: Evening cortisol		Mediator: Morning-to-evening slope	
	OR^a^	95% CI	OR^a^	95% CI	OR^a^	95% CI
Bullying						
Total effect^b^	1.85	1.47–2.33	1.85	1.47–2.33	1.85	1.47–2.33
Direct effect^c^	1.85	1.47–2.33	1.84	1.46–2.33	1.85	1.47–2.34
Indirect effect^d^	1.00	0.99–1.01	1.00	0.99–1.01	1.00	0.99–1.01
Mediated proportion^e^	0%	−1.5-1.4%	0%	−1.6-2.6%	0%	−1.9-1.6%

^a^Adjusted for gender, age, smoking, physical activity, alcohol consumption, body mass index, education, shift-work, awakening time, sampling time, round, and cohort. All measured at baseline

^b^Odds ratio of long-term sickness absence for bullied compared to non-bullied participants

^c^Association between bullying and sickness absence not mediated by cortisol levels

^d^Association between bullying and sickness absence mediated by cortisol levels

^e^Proportion of total effect that is mediated by cortisol levels

In the sensitivity analyses on frequently bullied (‘daily’ or ‘weekly’) participants we saw a similar pattern to that of the primary analyses. Frequent bullying was associated with sickness absence, but not with cortisol levels (data not shown).

The analyses of reverse causation showed that baseline cortisol levels did not predict subsequent workplace bullying among participants not reporting bullying at baseline (Table 5). We found no difference in cortisol changes from baseline to follow-up between participants with and without long-term sickness absence during the follow-up period (Table 6). We found that a history of sickness absence among non-bullied participants at baseline was associated with the risk of reporting workplace bullying at follow-up 2 years later (OR = 1.48, 95% CI = 1.05–2.08) (data not shown in table).

**Table 5:** Prospective associations of cortisol at baseline with workplace bullying at follow-up among participants not reporting bullying at baseline

	Bullied	Non-bullied	Crude OR	95% CI	Adjusted^a^ OR	95% CI
Morning cortisol						
Low (0–9.8 nmol/l)	72	1693	1	–	1	–
Moderate (9.9–15.8 nmol/l)	77	1758	1.03	0.74–1.43	1.02	0.73–1.42
High (15.9–100 nmol/l)	70	1862	0.88	0.63–1.24	0.83	0.58–1.18
Evening cortisol						
Low (0–1.0 nmol/l)	81	1839	1	–	1	–
Moderate (1.1–1.8 nmol/l)	63	1749	0.82	0.58–1.14	0.81	0.58–1.15
High (1.9–100 nmol/l)	88	2099	0.95	0.70–1.30	0.94	0.68–1.29
Morning-to-evening slope						
Low (−100–8.1 nmol/l)	63	1764	1	–	1	–
Moderate (8.2–14.2 nmol/l)	79	1684	1.31	0.94–1.84	1.42	0.99–2.01
High (14.3–100 nmol/l)	68	1626	1.17	0.83–1.66	1.26	0.87–1.82

^a^Adjusted for gender, age, smoking, physical activity, alcohol consumption, body mass index, education, shift-work, awakening time, sampling time, round, and cohort. All measured at baseline

**Table 6:** Changes in morning cortisol, evening cortisol, and morning-to-evening slope among participants with and without long-term sickness absence during a two-year follow-up period

	Changes in cortisol concentrations		Difference in the change in cortisol concentration between participants with long-term sickness absence and no-long term sickness absence (reference).			
	No long-term sickness absence	Long-term sickness absence	Crude difference	95% CI	Adjusted^a^ difference	95% CI
Morning cortisol^b^	2.25	2.36	0.11	−1.40-1.62	−0.38	−1.90-1.15
Evening cortisol^b^	0.39	0.31	−0.08	−0.49-0.33	−0.19	−0.61-0.22
Morning-to-evening slope^c^	−1.76	−1.85	−0.09	−1.60-1.42	0.21	−1.39-1.80

^a^Adjusted for gender, age, round, cohort, awakening time (baseline and follow-up), sampling time (baseline and follow-up) and changes from baseline to follow-up in: smoking, physical activity, alcohol consumption, body mass index, and education

^b^Changes in cortisol concentration (nmol/l) during the two-year follow-up period

^c^Changes in the difference between morning and evening cortisol (nmol/l) during the two-year follow-up period

## Discussion

We found no indication that cortisol mediated the association between workplace bullying and sickness absence. Although there was no support for our hypothesis that cortisol (as an indicator of the physiological stress response) is part of the causal pathway leading from workplace bullying to long-term sickness absence, we did find that high evening cortisol levels were associated with a decreased risk of subsequent sickness absence. We also found that workplace bullying was significantly associated with subsequent long-term sickness absence, and vice-versa. There were no significant associations between bullying and cortisol levels.

The results of this study regarding the association between workplace bullying and subsequent sickness absence were in line with previous findings [3–7]. In contrast, the few previous cross-sectional studies of workplace bullying and cortisol have indicated lower cortisol levels among those exposed to bullying [13–16], a result that was not replicated in this study. It is important to point out that an association has previously been shown between frequent workplace bullying and low cortisol concentrations in the WBH cohort [15]. The longitudinal associations workplace bullying and cortisol concentration have also previously been examined in the PRISME and WBH cohorts and changes in bullying status during follow-up was not related to changes in cortisol levels [38]. To our knowledge, no previous studies have examined the association between cortisol and sickness absence and no previous studies have looked at the reverse causal mechanism, i.e. whether long-term sickness absence is a risk factor of subsequent bullying, whether long-term sickness absence is associated with changes in cortisol levels, or whether cortisol levels predict subsequent workplace bullying.

There are several possible explanations for why we did not find the same association between workplace bullying and cortisol levels as the few previous cross-sectional studies. Cortisol concentration exhibits diurnal variation and the exact time of sampling could be important. We only measured cortisol concentration 30 min after awakening and at 20:00 h and thus were not able to calculate commonly used derived measures, such as the cortisol awakening response [39]. The few previous studies all collected additional samples and thus were able to examine the diurnal cortisol curve in more detail. It is possible that the association between workplace bullying and cortisol is not so straightforward as to be measurable with only two cortisol samples. For example, in the 2006 study by Hansen et al. it was primarily the bullied respondents’ lower cortisol concentration at awakening that contributed to the difference with non-bullied respondents [14]. We did not measure cortisol at awakening. The study by Kudielka (2004) used a different methodology and compared the diurnal cortisol rhythm of bullied participants and work days and days off, however, no comparison was made to non-bullied participants. In addition, Kudielka and Kern also used a different measure of workplace bullying [13]. The study by Hogh et al. (2012) also used a different measure of workplace bullying than our study, i.e. the revised Negative Acts Questionnaire, which may explain the different results [16]. The study by Hansen et al. (2011) used the same measure of workplace bullying as in our study and was based on the first round of the WBH cohort that also provides parts of the data used in this study and found an association with cortisol among only the most frequently bullied [15]. While we have performed analyses on the frequently bullied, frequent bullying is rare in our study population. In the PRISME and WBH cohorts there are only 32 and 13 participants that experienced bullying ‘daily’ or ‘weekly’, respectively. When examining the association between frequent bullying and cortisol levels we did not find any major differences compared to the results based on all bullied participants, but this could be a false negative finding caused by low statistical power due to the small number of frequently bullied participants (data not shown).

The analyses of reverse causation indicate that not only is workplace bullying a risk factor for long-term sickness absence, long-term sickness absence is also a risk factor for subsequent workplace bullying. This result puts emphasis on the importance of accounting sufficiently for the history of sickness absence, when examining whether workplace bullying is a risk factor of sickness absence. The analyses of reverse causation also showed that cortisol levels were not associated with bullying or sickness absence neither as risk factor nor as outcome.

### Strengths and limitations

Compared to the previous cross-sectional studies, this study is by far the largest as it includes 7451 observations and 5418 unique participants. We relied on a more thorough adjustment for awakening and sampling time than used previously, which could be important, since one of the strongest predictors of salivary cortisol concentration is the sampling time [23], and is thus a strong potential confounder if associated with workplace bullying or sickness absence, e.g. through sleep difficulties [40]. Additionally, we have obtained registry information about long-term sickness absence for the entire follow-up period as well as the two year prior to baseline and excluded all participants with a history of long-term sickness absence to decrease the risk of reverse causation.

Both cohorts had a low baseline participation rate. Among the invited, only 49% of the WBH cohort and 45% of the PRISME cohort participated in the study, which could have affected the validity of our study due to differential participation. At follow-up, the participation rate was higher than at baseline, but selection may still have biased our findings. Participants exposed to workplace bullying or with low cortisol levels were less likely to participate in the follow-up rounds. Non-participants also had an increased risk of sickness absence compared to the participants of the study (data not shown). The fact that non-participation was associated with both workplace bullying, cortisol levels and sickness absence could have caused biased results toward no associations.

By relying on self-reported measures of workplace bullying we obtain the best estimate of the associations related to the *perception* of being bullied. However, our results may be inflated by reporting bias due to underlying factors, such as personality or illness, increasing both the perception of bullying and the risk of sickness absence [19].

Due to the available register data, this study only considers spells of 30 days or more to be long-term sickness absence. Consequently, our outcome measure did not capture transient health problems, and we could not examine the association between workplace bullying and short-term sickness absence. It is possible that cortisol is part of the causal pathway leading from workplace bullying to short-term sickness absence.

Since we found no indications that cortisol is part of the biological mechanism linking workplace bullying to long-term sickness absence, this negative finding is unlikely to be an artifact caused by us measuring bullying and cortisol at the same time. While such concurrent measures could be a source of bias due to reverse causation, we have no reasons to believe that the cross-sectional analyses have inflated the true association, when we find no association between the measures.

We also have to consider the risk of a false positive finding. We found no association in all but one of the many analyses we performed. High evening cortisol levels were associated with a decreased risk of subsequent sickness absence, but the upper confidence limits were close to unity. Thus, this single significant association should be interpreted with caution, particularly as the remaining results do not support an association between cortisol and sickness absence.

Additionally, we used a simple measure of the cortisol levels that was based only on morning and evening cortisol levels. Since cortisol has a substantial diurnal variation [23] and many different measurement strategies have been suggested [39], we cannot rule out that a more complex association exists which would require a more complex measurement strategy. In future research there may be a need to assess the physiological reaction of the participants more thoroughly, for instance by a) collecting multiple daily cortisol samples to better reflect the diurnal variation of cortisol, b) collecting cortisol at several instances to assess changes over time, or c) relying on other measures of the physiological stress reaction, such as allostatic load or heart rate variability.

## Conclusion

In conclusion, this study suggests that there is no straightforward and simple association between cortisol and long-term sickness absence and that the association between workplace bullying and long-term sickness absence was not mediated by cortisol. Thus, we found no support for our hypothesis that changes in cortisol levels reflect a biological mechanism linking workplace bullying and long-term sickness absence.

## Abbreviations

CI: Confidence interval
HPA: Hypothalamus-pituitary-adrenal
LTSA: Long-term sickness absence
OR: Odds ratio
PRISME: Psychosocial RIsk factors for Stress and MEntal disease
RSS: The Danish register of sickness absence compensation benefits and social transfer payments
WBH: Workplace Bullying and Harassment
